# Implementing a singing-based intervention for postpartum depression in Denmark and Romania: a brief research report

**DOI:** 10.3389/fmed.2023.1249503

**Published:** 2023-12-20

**Authors:** Katey Warran, Calum Smith, Hanna Ugron, Oana Blaga, Nicolai Lund Ladegaard, Louise Frøkjær Carstens, Lucy Nicholls, Alexandra Burton, Rarita Zbranca, Mikkel Ottow, Daisy Fancourt, Nils Fietje

**Affiliations:** ^1^Social Biobehavioural Research Group, Research Department of Behavioural Science and Health, University College London, London, United Kingdom; ^2^Behavioural and Cultural Insights Unit, World Health Organization Regional Office for Europe, Copenhagen, Denmark; ^3^Nuffield Department of Population Health, University of Oxford, Oxford, United Kingdom; ^4^Centrul Cultural Clujean, Cluj-Napoca, Romania; ^5^Center for Health Policy and Public Health, College of Political, Administrative and Communication Sciences, Babes-Bolyai University, Cluj-Napoca, Romania; ^6^Department of Affective Disorders, Aarhus University Hospital—Psychiatry, Aarhus, Denmark; ^7^Department of Clinical Medicine, Aarhus University, Aarhus, Denmark; ^8^Den Kreative Skole, Region Midtjylland (Central Denmark Region), Viborg, Denmark; ^9^Region Midtjylland (Central Denmark Region), Viborg, Denmark

**Keywords:** art and health, culture and health, postpartum depression, implementation, feasibility

## Abstract

**Background:**

There is a burgeoning evidence-base that demonstrates the positive impact of the arts on our health, wellbeing, and health behaviors. However, very few studies have focused on how to optimize the implementation of these activities for different sociocultural contexts. Due to the increasing interest in scaling effective arts interventions as part of public health strategies, and in line with global goals of achieving health equity, this is an essential focus.

**Aim:**

Using the case study of a singing for post-partum depression (PPD) intervention with empirically-demonstrated clinical effects, this brief research report explores implementation of an arts and health intervention that has been successful in the United Kingdom (“Music and Motherhood”) for the new contexts of Silkeborg (Denmark) and Cluj-Napoca (Romania).

**Methods:**

Data was collected from participants at all levels of the implementation structure including at local and management levels. The study draws on qualitative implementation data to explore participant experiences, including one-to-one interviews, written testimonies, meeting minutes, ethnographic researcher reflections and focus groups, including data from 46 participants in total.

**Results and conclusion:**

We explore implementation and adaptation across five key themes: (1) acceptability and feasibility; (2) practical and structural barriers and enablers; (3) adoption and sustainability; (4) broader contextual factors affecting implementation and sustainability; and (5) project structure and processes. Taken together, the themes demonstrate that arts interventions need to be adapted in culturally sensitive ways by stakeholders who have local knowledge of their environments. This research serves as an informative foundation for use by other researchers that aim to expand the reach and impact of arts-based interventions.

## Introduction

As the evidence on the role of the arts in improving health and wellbeing and promoting health behaviors has grown (1–4) so too has the interest from policymakers, and health and social care commissioners regarding how to improve access to the arts as a form of public health provision (e.g., social prescribing initiatives) (5, 6). Global policies demonstrate increasing commitment to providing infrastructures that enable the growth of arts interventions to support public health challenges (5), and the World Health Organization (WHO) has acknowledged the importance of the arts to achieving global development goals (7). The growing momentum in this area has also spurred the creation of several key national and international bodies (4). Yet, despite the increased attention given to the structures surrounding the delivery of arts and health interventions within policy and practice, very little research has focused on how to optimize and evaluate implementation or how certain evidence-based interventions may need to be adapted for new contexts. The latter is particularly important in view of the growing recognition of the need to scale-up effective health interventions to improve access, rather than focusing solely on new innovations that may never be implemented in real-world settings (8). Whilst it is indeed still a priority to improve the quality of the evidence in arts and health (9, 10) and to explore a range of different epistemological perspectives that can better acknowledge the complexity of the arts as culturally-embedded, relational activities (11), there is a clear gap to explore the feasibility of scaling well-evidenced interventions.

The few studies that do exist exploring implementation of arts interventions include a scoping review exploring implementation of online singing groups for people living with dementia (12), a randomized controlled trial exploring the feasibility and acceptability of music therapy in managing delirium (13) and a study that developed an implementation plan in the context of depressive symptoms in young adults (14). A selection of implementation guides are also available documenting how to replicate music therapies within similar settings (15–17). Few of these guides, however, consider the role of cultural context in implementation. This is important in view of the “complex” nature of the arts (18), health, and health behaviors (19), whereby socioeconomic, cultural and political factors can be viewed as moderating the relationship between the arts and health (20). Work by Belgrave and Kim (21) provides music therapists with the tools to integrate understanding of different cultural and social identities, such as heritage, age, and health beliefs, into their practice, but broader organizational and socioenvironmental factors are left unexplored. Although these guides provide specific support for music therapists, research is required in order to develop implementation guidance for community arts practitioners (i.e., not therapists), and to explore the organizational structures surrounding delivery and implementation of interventions in different sociocultural contexts.

Using the case study of a singing for post-partum depression (PPD) intervention, this study explored how an arts and health intervention that has been successful in the United Kingdom [“Music and Motherhood” (22)] was adapted to new contexts in Silkeborg (Denmark) and Cluj-Napoca (Romania), with a view to providing support and guidance on future implementation and scale-up of arts interventions.

## Materials and methods

### Context

#### Music and Motherhood

A singing for PPD intervention known as “Music and Motherhood” was selected to be implemented in Denmark and Romania following a roundtable discussion of evidence-based arts and health interventions amongst the study team including the behavioral and cultural insights (BCI) Unit at WHO Regional Office for Europe, arts, third-sector and health organizational representatives and researchers (*n* = 17). The original Music and Motherhood randomized controlled trial compared the effects of a 10-week singing and play intervention on symptoms of PPD for new mothers, with results showing that the intervention reduced PPD symptoms by 38% and led to faster recovery in mothers with moderate–severe symptoms when compared to usual care (2, 23). It was selected based on evidence that shows singing-based interventions to be beneficial for PPD, alongside the challenges of there being no complete treatment solution (24, 25).

This single-arm feasibility study drew on the original protocol for Music and Motherhood (22). We sought to have a dual focus to explore the feasibility of implementation and to evaluate the perceived impact of the intervention. The intervention consisted of two 10-week singing interventions in Denmark and Romania, with the groups in Romania consisting of one Romanian-speaking group and one Hungarian-speaking group, as both languages are spoken within the region.^1^

In order to support the localization of the intervention, patient and public involvement (PPI) groups were organized in each of the locations. These groups were comprised of mothers with lived experience of PPD, musicians, researchers, and health specialists. The PPI groups met up to three times during the preparatory phase, advising on issues such as location, timing, music choices, and safeguarding.

### Participants

Data was collected from participants at all levels of the implementation structure including at local (e.g., new mothers with symptoms of PPD, singing leads, in-country project managers, healthcare staff, referrers) and management (e.g., WHO, University College London) levels (see Table 1).

**TABLE 1:** Number of participants per data collection method*.

	Mothers Denmark	Local staff Denmark*	Mothers Romania	Local staff Romania**	Core study management (see Table 2)
One-to-one interviews	*N* = 3		*N* = 4	*N* = 5	*N* = 1
Written testimonies	*N* = 2		*N* = 3	*N* = 4	
Focus groups	*N* = 7 (2 groups)	*N* = 4 (1 group)	*N* = 12 (2 groups)		*N* = 9 (1 group)

*Please note that some participants engaged in multiple data collection methods. The total number of participants included was 46 (Denmark *n* = 17, Romania *n* = 19, Study management *n* = 10).

**Local staff includes singing leads, healthcare staff, referrers, venue managers and project management.

**FIGURE 1:**
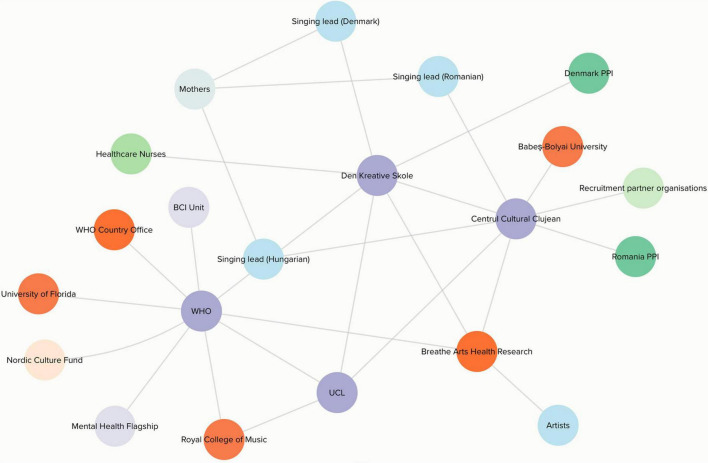
Stakeholder map. Circular nodes in this map represent individuals and/or organizations, and lines connecting these nodes represent collaborative working relationships/knowledge sharing, or project input. Nodes in purple represent project management organizations, nodes in orange represent research/session support and input, nodes in light blue represent artistic input, nodes in mint green represent recruitment, nodes in darker green represent PPI, nodes in lilac represent internal institutional support, the node in grey represents mothers who sang in our singing groups, and the node in light orange represents the funder.

Recruitment of partner organizations who led on in-country implementation was facilitated by the BCI Unit at the WHO Regional Office for Europe and drew on pre-existing arts and health networks. Organizations were approached based on their previous successful delivery of community-based interventions (Romania) or arts and health projects (Denmark). The Social Biobehavioural Research Group at UCL joined as a partner, building on their status as a WHO Collaborating Centre for Arts and Health. Based on these initial partners, recruitment happened organically, as illustrated in Figure 1. In Denmark, recruitment of mothers for the singing groups happened through referral from the healthcare system (e.g., via health nurses). In Romania, recruitment involved working with multiple cultural, community and health organizational partners.

### Methodology and methods

This study is part of a broader mixed-methods project exploring implementation and effectiveness of singing groups in different cultural contexts (26). For this article, we report findings from our analysis so far, drawing on qualitative implementation data to explore participant experiences. This included one-to-one interviews, written testimonies, meeting minutes, ethnographic researcher reflections and focus groups (see Table 1).

All mothers were invited to participate in focus groups at the end of the study and to provide additional feedback in the form of a written testimony. Based on availability, some mothers only provided written feedback. A smaller selection of mothers (*n* = 3–4 per country, the first who volunteered) were additionally invited to participate in a one-to-one interview to further explore feasibility of implementation. All members of the management structure were invited to participate in a focus group or an interview (based on availability), and singing leads were invited to participate in a one-to-one interview. Participant numbers are included in Table 1.

Interviews or focus groups with local staff were conducted by HU in Romania and NL in Denmark. Focus groups with mothers were conducted by OB and a mental health specialist in Romania and by LC in Denmark. Written testimonies were collected via email. Meeting minutes from bi-weekly online study team meetings (see Table 2) were created by CS. Ethnographic reflections were created by KW at analysis meetings, and the management focus group was conducted by AB, as someone not directly involved with implementation (see Supplementary Appendix example Topic Guide). Given the importance of our team structure to explaining our implementation processes, CS used Kumu (software) to create a stakeholder map (see Figure 1).

**TABLE 2:** Role in the project of those participating in the core study management who were part of a focus group or interview about implementation*.

Category**	Number of participants
Local Denmark management team	2
Central management team–Denmark	1
Central team	3
Local Romania management team	3
Central management team–Romania	1

*One participant was unable to participate in the focus group so a one-to-one interview was conducted. We have not highlighted who this was to preserve anonymity.

**Local teams are those on-the-ground who were part of in-country implementation. Central teams are those who were involved with strategic decisions and management, but who were not part of logistical delivery in-countries. Further details are not provided to preserve anonymity.

### Analysis

Data was collected in Danish, English, Hungarian or Romanian. Discussions were transcribed using transcription agencies, software or by hand and analyzed using Framework Method (27) by OB (Romania), NL (Denmark), and KW (United Kingdom—management data). NL conducted analysis by hand, and OB and KW used NVivo 12. The framework used to guide analysis drew on theories from implementation science (28–31) (see Supplementary Appendix). Although a deductive approach to analysis was taken, this was supplemented with open, inductive coding to account for unexpected findings. Once coding was complete, codes were translated into English and discussed between KW, OB, and NL at a series of analysis meetings. Themes were constructed out of codes by KW in view of group discussions and through searching for patterns across the translated data from all countries. The discussions also included reflection on researcher positionality and the sociocultural contexts of data collection. Themes were constructed out of codes by KW in view of group discussions and through searching for patterns across the translated data from all countries (see Table 3 for an overview of themes, sub-themes and descriptions).

**TABLE 3:** Themes constructed from the analysis procedure showing processes for adapting and implementing the Music and Motherhood intervention.

Theme	Sub-theme	Description
1. Acceptability and feasibility of the singing intervention	1.1. Suitability of content and structure	The choice of songs and structure of the classes were appropriate, especially in view of including babies and constructing “a group” that provided consistent support.
	1.2. Suitability of project team	The people organizing the intervention were important to its success, with particular emphasis placed on the singing lead as playing a crucial supporting role.
	1.3. Suitability for supporting PPD symptoms	The intervention was perceived to support the mental health of mothers with PPD, but additional psychological supports could be considered alongside the classes.
2. Barriers and enablers	2.1. To mothers’ participation in the intervention	There were some structural challenges to attending for mothers, but these didn’t prevent attendance. One factor in this could be that our participants enjoyed singing.
	2.2. To implementing the intervention	Implementation barriers/enablers existed at (1) on-the-ground and (2) strategic management levels, including in relation to staff training and flexible economics.
	2.3. To conducting the research	It was perceived that the research burden for participating in the study was high, but there were mixed views from health nurses in relation to the ease of the process.
3. Adoption and sustainability of the singing programme	3.1. Potential changes to the intervention	Overall, the intervention design was perceived as appropriate but there were suggestions of having more time for socializing and making it longer in the future.
	3.2. Need to build infrastructure	Resources, time and new referral pathways were expressed as being needed to ensure the longer-term sustainability and scalability of the intervention.
4. Broader contextual factors affecting implementation	4.1. Cultural and social factors	Cultural constructions of “PPD,” “healthcare,” and “research” differed between Romania and Denmark which affected the interventions’ delivery and sustainability, alongside the societal uncertainty and restrictions of the COVID-19 pandemic.
	4.2. Systemic issues	Referral and recruitment challenges were experienced due to systemic issues, with ethical approval systems also impacting the timeline of intervention delivery.
5. Project structure and processes	5.1. Unique organizational collaborations and responsive structures	The structure of the project team comprised of multiple levels of management, including on-the-ground partners, intersectoral collaborations and cross-country strategic management that operated as “a whole kind of machinery.”
	5.2. Delivery of the training programme	Training provided by Breathe supported with practical implementation and provided energy and enthusiasm. Research training from UCL was also valuable.

## Results

### Theme 1: Acceptability and feasibility of the singing intervention

The suitability of the content and structure of the singing intervention (subtheme 1.1) was important to acceptability and feasibility. In Romania, mothers enjoyed the songs that “varied in style, energies and cultural backgrounds” and the “body movement and voice exercises” (Mother Romanian Group ID2). However, one participant remarked that “it would have been easier if the lyrics were sent after each session” (Mother Danish Group ID4). The suitability of the content was also expressed in Denmark. One mother noted that the “songs make you reflect and think” (ID4) and referrers described “being together in song” as “a great idea” (Denmark Local Staff ID1), with “the fact that they can bring their child” noted as “significant” because “they don’t have to think about childcare” (Denmark Local Staff ID1). Across both countries, the importance of the intervention as “a group” was voiced. The group created “an atmosphere of acceptance” (Mother Romanian Group ID5), “companionship with others” (Denmark Local Staff ID1), “a feeling of belonging” (Mother Danish Group ID5), and a “space” where mothers could “meet around something you already know is vulnerable” (Mother Danish Group ID2). The classes also provided a routine and consistent support. One mother noted “it’s something I have had a countdown on” (Mother Danish Group ID6) in relation to counting down the days between the sessions, with referrers also noting there was “something predictable about it” which was “especially important” for participants (Denmark Local Staff ID1). The value of consistency and “the group” have also been highlighted in previous research exploring singing for PPD (32), and additionally align with broader theories spotlighting the importance of group-level factors to health and wellbeing. Notably, the social cure approach (which combines social identity theory and self-categorization theory) suggests that meaningful identification with a group provides psychological resources that support health (33), and this too can be applied to the context of singing (34).

Another key aspect was the suitability of the project team (subtheme 1.2). One mother in the Romanian group noted that the success of the intervention “was primarily down to the people” with the project manager and singing leader “open” and “supportive” (Mother Romanian Group ID2). This participant also described how these project team members shared “their own experiences as mothers” (Mother Romanian Group ID2) which contributed to the classes being perceived as “authentic,” suggesting that sharing experiences across mothers and staff created an inclusive group environment. Across both countries, the singing leaders were described as important to the acceptability of the intervention. The singing leader “established an open and welcoming space” (Mother Danish Group ID1), went “above and beyond expectations” (Mother Danish Group ID1), was a “fabulous teacher… because she is so… human… she also shares herself” (Mother Danish Group ID7) and expressed an “interest in one’s child” (Mother Danish Group ID8). This highlights the importance of the soft skills of the singing lead (e.g., relationship-building) as crucial to the success of the intervention, alongside musical and leadership abilities, as has been documented elsewhere (29, 35).

The final sub-theme here explores the suitability of the singing intervention for supporting symptoms of postpartum depression (subtheme 1.3). Participants found the classes to be “useful and beneficial for their mental health” (Romania), they helped to “hold on to things that bring joy and wellbeing” (Mother Danish Group ID1), “reconnect with my body” (Mother Danish Group ID6), and address difficult emotions through feelings being “mirrored and held” (Mother Danish Group ID2) as well as supporting mothers to “function better in daily life” (Mother Hungarian Group ID8). One mother also noted that the WhatsApp group created for those participating had “a good impact on my mental state” (Mother Romanian Group ID2). However, one mother expressed that she “expected some direct method to improve mood,” saying that the singing group alone was “not enough” and therefore choosing to see “a support specialist” too (Mother Hungarian Group ID8). Whilst this could be considered for future adaptations, “some participants may be hesitant to join [the classes] due to the fear of being open about their mental health struggles” (Romania Local Staff ID2), suggesting that a greater emphasis on mental health could create barriers. In Denmark, it was recommended that future interventions “include mothers with less severe symptoms” which may help the intervention to play a “preventative” role (Denmark Local Staff ID1). Given that the mother who expressed wanting more “direct” help also sought additional support, one option for future adaptation of the programme could be to further expand complementary psychological support options available.

### Theme 2: Practical and structural barriers and enablers

There were specific practical and structural barriers and enablers to mothers’ participating in the classes (subtheme 2.1). The practicalities of attending the classes were viewed as barriers, such as the time of the classes which involved changing the sleep schedule of babies (“my son didn’t sleep, it was exhausting to go,” Mother Hungarian Group ID8) and transportation (“It’s the logistics that’s a challenge… the bus and stuff… it’s a frustration” Mother Danish Group ID1; “difficult to find parking… almost gave up” Mother Danish Group ID4). However, mothers also noted that these factors didn’t prevent them from coming, with minimizing disruption to “daily routines” taken into consideration when “scheduling the sessions” (Romania Singing Lead ID1). Referrers in Denmark perceived that “the fact that they can bring their child is significant” in enabling participation because “they don’t have to think about childcare,” which was also supported by “the fact that it has been free” (Denmark Local Staff ID1). Another key enabling factor was the enjoyment that mothers and their babies derived from singing and their willingness to attend, with one noting that the reason they joined was because they “love to sing” (Mother Danish Group ID1) and another that the sessions are also “enjoyable for one’s child” (Mother Danish Group ID4). However, the singing lead for the Hungarian group also felt that there were “language barriers” to having a Hungarian-only speaking group, as Romanian-speaking mothers could not join (Romania Singing Lead ID2).

Practical and structural barriers and enablers to implementing the intervention (subtheme 2.2), operated at two levels: (1) in-country implementation and (2) strategic project management. In relation to the former, this included “the lack of communication with the public health sector and the absence of recommendations from healthcare providers” (Romania Singing Lead ID1) and “a lack of structured referral pathways” (Romania Local Staff ID1). In Denmark, whilst there were formal referral pathways, barriers were seen in relation to healthcare staff training and not having enough experience of what the intervention would entail. Participants reflected that there should have been a “trial run [of singing] at the staff meeting” (Denmark Local Staff ID1) because it is “important to have experienced it [singing] to be able to sell it to mothers” (Denmark Local Staff ID1). In Romania, enablers to implementation included “involvement of a PPI group” to ensure suitability, as well as “a well-designed communication strategy, including the use of sensitive and conscious language” for recruitment (Romania Local Staff ID1). At the strategic level, it was noted that “being able to move around money has been important” (Study management ID2), with the profile of WHO also viewed as an important enabler (“an institution where doors open once you show your badge,” Study management ID4), giving the project “real credibility” (Study management ID3).

Practical and structural barriers and enablers to conducting the research (subtheme 2.3) also intersected with these implementation factors. At the strategic level, it was suggested that the research burden for the project was quite high, notably affecting mothers joining the project (“research activities were preventing someone from actually joining the intervention,” Study management ID2; “I know they [mothers] were worried about the scales,” Study management ID8), and a participant from the Romanian group stated that the “long text [study description] was very discouraging” (Mother Romanian Group ID5). Nonetheless, there were mixed views of the research burden in Denmark. One health nurse noted that they didn’t have to spend very much time with mothers when recruiting because the screening process was “not very long” (Denmark Local Staff ID2), but another stated that form filling was difficult (“the mothers couldn’t handle it,” Denmark Local Staff ID2). Yet, these nuances of research burden were mentioned less by the mothers themselves. One reason for this could be because “most of the participating mothers are those that understand research” and have “higher education” which could be “a source of exclusion for others” (Study management ID6). Future research could explore further whether research literacy and burden are reasons for not signing up to an intervention such as this is.

### Theme 3: Adoption and sustainability of the singing programme

This next theme explores the potential adoption and sustainability of the intervention. Overall, participants noted that they would “recommend it [the intervention]” (Mother Hungarian Group ID8), with referrers in Denmark expressing a strong desire to continue delivering the sessions (“I definitely want that [the classes] to continue” Denmark Local Staff ID3). However, some participants suggested potential changes to the intervention (subtheme 3.1) for it to be sustainable in the longer-term. Of note, mothers from the Hungarian group remarked that they would have liked more socializing, also including more opportunities to “interact” with their babies (Mother Hungarian Group ID14). Yet, this could be unique to this group for logistical reasons because the mothers couldn’t stay in the room after the class finished, in contrast to the Romanian group who could stay and engage in “chit-chat” (Ethnographic Notes). This also supports the rationale for the importance of fostering a sense of “the group” as a mechanism for improved health, as explored in Theme 1. A mental health specialist in Romania also suggested a longer intervention, which was supported by mothers in Denmark who stated that it was hard when the group ended because the classes “completely disappeared from your life” (Mother Danish Group ID1) and that it was hard to “close the wound” in relation to emotions that had been brought out due to engagement with the classes (Mother Danish Group ID9).

From a management perspective, it was viewed as important “to build an infrastructure around these kinds of arts and health initiatives” (Study management ID7) to ensure they can be fully adopted within the contexts of Denmark and Romania (subtheme 3.2). “Passion” was a key part of what made this project work within a setting of limited resources (“money was quite modest” Study management ID10) and time (“I don’t actually have a lot of time to spend on this project” Study management ID7). This could be considered a problematic structure when thinking about longer-term sustainability because it is reliant on passionate individuals and creates a precarious foundation. Resources also vary across the countries. In Denmark, the team have secured funding to “keep on doing this for another two to three years” (Study management ID2), whereas Romania haven’t. In the latter case, the focus is now on “trying to continue momentum” and working toward the creation of “a social prescribing mechanism” (Study management ID7). The current structures were described as “guerrilla activity” (Study management ID7), and therefore not sustainable in the longer-term, with it suggested that WHO needs to explore how to “advertise [the intervention] to Member States” to build the infrastructure needed (Study management ID7). Further, in Romania, it was noted that the context of Cluj-Napoca would be very different to smaller towns in Romania which have less services and “urban infrastructure” (Study management ID10). This mirrors wider research on social prescribing which argues that implementation varies in different countries, reflecting local, cultural, healthcare and political contexts (36).

### Theme 4: Broader contextual factors affecting implementation and sustainability

Cultural and social factors (subtheme 4.1) affected implementation. A project manager from Romania expressed that their context is “complex” because there’s “a culture in Romania and an Eastern European ethic of feeling that the research is the ‘price you have to pay’ to participate” (Ethnographic Notes). Because the classes were free, there may have been a sense that research participation was the “trade-off” for receiving the classes. In Romania, PPD was considered “a taboo subject” with motherhood depicted in a way where the “mother is just this perfect, perfectly happy being” (Study management ID10) and many “underestimating their need for help” (Romania Local Staff ID1), with a lot of “stigma surrounding mental illness” (Romania Local Staff ID3) and “postnatal depression” (Romania Local Staff ID1). This reflects broader literature that argues PPD in Romania is often “ignored” and considered “not a disease” (37). This was reinforced by the suggestion that “Mothers do not trust the healthcare system [in Romania] and that there is anger and negativity toward birth experiences” (Ethnographic Notes). In view of this distrust, it is possible that integration into formal healthcare pathways may be more challenging than in other cultural contexts, as mothers may be less inclined to join a programme relating to mental health through healthcare services. By contrast, mothers in Denmark were directly referred by health nurses for this study, with mothers describing this as a reason for joining, “It was actually because of a 2-month examination with the health visitor… and I thought, yeah let’s just give it a try” (Mother Danish Group ID1).

The social context of COVID-19 also affected intervention delivery. In Romania, due to the low uptake of COVID-19 vaccination, it was decided that the classes would run into the summer to ensure that they could take place in a semi-outdoor location. However, many mothers experienced “scheduling conflicts” because of it being the summer which “made regular attendance challenging” (Romania Singing Lead ID2). Further, it was noted by a member of the strategic management team that “post COVID” and “with Romania neighboring Ukraine,” it is an important time for support to have a “mental health angle” (Study management ID10). This aligns with broader sentiments reflected in the European Commission comprehensive, prevention-oriented and multi-stakeholder approach to mental health (38).

There were also systemic issues (subtheme 4.2). In Romania, it was noted that “the intervention faced various challenges related to the medical system, such as a lack of structured referral pathways and the need for systemic and structural changes” (Management, Romania). Whilst Denmark did follow a referral process for recruitment, there was low uptake and it was hard for healthcare staff to manage multiple priorities. Although, it was hypothesized that this could have been due to the project being a research study, rather than an issue with the referral process itself (“suspect it is a problem with research elements involved”; Meeting Minutes). The Institutional Review Board (IRB) was also described as “a complicated one” that “was a hassle to go through” (Study management ID9), with the processes pushing back the timeline for the project (“I think it took one year,” Study management ID10). However, it was also noted that “the protocol was actually bettered” (Study management ID10) through the ethics process. For this study, it took extra time to build the protocol to undergo ethical approval as it was the first arts and health study led with the BCI Unit at WHO Regional Office for Europe. As this intervention is implemented more widely, our findings highlight a need to build on the foundations created here to set realistic timelines and ensure approvals happen smoothly.

### Theme 5: Project structure and processes

The project was viewed as having unique organizational collaborations and responsive structures (Subtheme 5.1), comprised of multiple levels of management, including on-the-ground partners and PPI groups, intersectoral collaborations and cross-country management (“a whole kind of machinery,” Study management ID7). Fundamental to the structure was the involvement of reputable organizations, with it suggested that “more institutions that are internationally renowned for their high-quality work” would be needed to scale this intervention up in the future (Study management ID4). There was an iterative element to the structure (“kind of doing it as we go along,” Study management ID5) which meant that the project was more demanding than expected (“don’t think that we actually understood what we were getting into” Study management ID2). Yet, it was suggested that having a more fixed structure could mean being “stronger in certain areas” but not able “to develop this (as) freely and as focused as we (have)” (Study management ID7). Having access to different kinds of resources (“human resources available” Study management ID10) and networks were also important (“rich network of professional health, non-health [and] cultural contacts,” Study management ID10), with the study team itself described as a “network of trust” (Study management ID6). Further, the PPI group was “essential in guiding the program and maintaining contacts with its members and informal networks” that were “crucial for its [the intervention’s] success” (Romania Local Staff ID1).

The training delivered (Subtheme 5.2) to project staff and singing leaders (2 separate workshops) by United Kingdom organization Breathe Arts Health Research and research training provided by UCL was important to implementation. The training from Breathe supported with what “to prepare for,” including how to create “the right atmosphere” (Study management ID6) and make music decisions (“[the song from the training] ended up being sung,” Study management ID5). Participants viewed Breathe as “heroes” (Study management ID2) because they were renowned in delivering the intervention, with the training “very motivational” and “inspiring” (Study management ID8). The training helped to make the project feel “real” (Study management ID7, FG) and less theoretical (“it suddenly became not a protocol,” Study management ID5). Although delivering training from an organization based in the United Kingdom to support delivery in different cultural contexts could be perceived as a “top-down” approach, participants felt that “coming from UK” didn’t have any “judgment value attached” (Study management ID6) with it “mak(ing) it bigger somehow” (Study management ID2). The training provided an emotion-setting role in making participants feel “fueled [with] enthusiasm” and “grateful” (Study management ID8) for the training received. The research training provided by UCL was viewed as “helpful” for “collecting data” (Study management ID6) and “valuable also for the future” (Study management ID8).

### Reflections on the process

Comparing the process of adaptation to the two sites in Denmark and Romania, three areas stand out: logistics; recruitment; and language. In both countries, discussions (informed by the PPI groups, as well as within and between the management team and local implementation groups), led to logistical choices that differed between the Danish, Romanian, and Hungarian singing group sessions. These differences included the choice of locations for the singing groups, timings for when they took place; and the logistical support offered for mothers to attend. Recruitment was also very specifically localized to suit the health systems realities of the participating countries. Although the recruitment in Denmark was linked to the health system through a process of referral from health nurses, challenges did become apparent. Since the referral process was not formally embedded, and because the nurses were not uniformly familiar with the intervention, referrals were slow at first, until a workshop was organized. This allowed the health nurses to better understand the intervention, and to champion it in their practice. In Romania, recruitment was fundamentally different, and relied on advertising and campaigning, both analog (for instance through printed posters in relevant doctor practices) and virtual (through social media and online influencers). Finally, an important concern prior to the project had been the question of linguistic translation. The implementation teams at all levels and in collaboration with the PPI groups paid particular attention to localizing the choice of music and the way the intervention was described. While this was an important part of the implementation process, it is worth pointing out that language was not in fact as much of a barrier as originally anticipated. Significant similarities existed in the way language was deployed to de-medicalise the intervention (e.g., by not using terms equivalent terms to “depression” in the different languages), even if the process of arriving at these conclusions differed (for instance, in the Romanian context, and important argument for de-stigmatizing the word “depression” questioned the utility of softening the language around the Music and Motherhood intervention). Regarding the choices of music used during the sessions, these were of course adapted to the different language settings. Nevertheless, because all singing leads had received the same training, which had introduced them to songs that are not language based, some song choices were actually the same across all groups.

## Conclusion

This study demonstrates that an evidence-based arts and health intervention (“Music and Motherhood”) can be adapted in culturally-sensitive ways to support populations beyond the original context in which it was developed. It serves as an informative foundation to expand the reach and impact of arts-based interventions in the future. However, cultural and systemic factors need to be considered when thinking about longer-term sustainability, such as in relation to the stigma associated with mental health conditions such as PPD and (dis)trust of healthcare systems that may differ in different countries, alongside the broader changing sociopolitical landscape. Integration of arts interventions into public health needs to consider what infrastructures are suitable to sustain arts and health interventions based on specific local country needs and considerations. Future research should also explore how to adapt this intervention for other country contexts, particularly in view of the challenges and learnings highlighted here (e.g., flexible economic structures, need for passionate teams, IRB processes), with the study team already exploring how to do this through scaling the intervention to Italy.

## Data availability statement

The datasets in this article are not publicly available to preserve the anonymity of participants. Requests to access the datasets should be directed to KW, k.warran@ucl.ac.uk.

## Ethics statement

The studies involving humans were approved by the Ethics Review Committee of the World Health Organization and the National Ethics Committees in Romania and Denmark. The studies were conducted in accordance with the local legislation and institutional requirements. The participants provided their written informed consent to participate in this study.

## Author contributions

KW, CS, and LN drafted the report for this manuscript, with specific feedback from NF, AB and DF. KW led on the design of the methodology for this specific manuscript and the overall study data collection, and NF led on the overall supervision of the project, including funding acquisition. HU and RZ provided specific advice on the Romanian context, and LC and MO provided advice on the Danish context. NL and OB led on the in-country analysis of data and provided summaries of analysis in English for discussion which formed the foundation of themes for this article. All authors contributed to the conception and design of the study (except AB who joined the team after conducting the focus group for the management team), and all authors approved the final manuscript for publication.

## Footnotes

1 Hungarian-language speakers are the largest minority group in Romania.
