# Supplementary material for: Implementing a singing-based intervention for postpartum depression in Denmark and Romania: a brief research report
Source: Front Med (Lausanne). 2023 Dec 20;10:1249503. doi: 10.3389/fmed.2023.1249503 (PMC10769490; doi:10.3389/fmed.2023.1249503)
Supplement: Supplementary file 1 [file Table_1.DOCX]

**APPENDIX**

1. **Framework used to analyse implementation data**

| **Reasons for taking part** – why mothers decided to join |
| --- |
|  |
| **Acceptability/appropriateness** – whether the singing programme is perceived to be agreeable/acceptable/relevant for management of post-natal depression (PND) e.g., suitability of content and structure, appropriate singing leader etc. |
|  |
| **Feasibility** – whether it was feasible for participants to attend the singing programme to support PND symptoms e.g., willingness to attend, anything that prevented attendance |
|  |
| **Intention to adopt** – whether participants intend to continue aspects of the classes now that they are over (e.g., singing exercises, meeting other mums) and anything that may prevent or enable adoption of these behaviours |
|  |
| **Fidelity of receipt** - extent to which programme was received as it was intended e.g., how easy/difficult did participants find it to engage, any changes asked to be made to the singing programme e.g., not bringing their babies |
|  |
| **Unintended consequences** – Any positive or negative consequences that were not anticipated at the start of the project |
|  |
| **Associated costs** – Any costs incurred for mothers as a result of intending (includes indirect costs such as time off work, extra childcare, travel costs) or extra resources required/time from project partners or referrers |
|  |
| **Sustainability** – any facilitators or barriers to sustained delivery of the singing programme e.g., is there a willingness to continue supporting it (e.g., from partners) or referring mothers into it (referrers) |
|  |
| **Implementation strategies** – were any strategies used to deliver and implement the programme, such as methods or techniques used to enhance and promote adoption, implementation or sustainability of the programme e.g., strategies used to identify and refer patients into the singing classes |
|  |
| **Training** – if training was received, is there any feedback on this |
|  |
| **Key learnings** – any reflections on learnings from the project |
|  |
| **Adaptations** – did the singing programme have to change in any way for its delivery e.g., the length of the classes, the location, the singing leader |
|  |
| **Contextual or structural factors** – any other factors that may have created or been facilitators or barriers to the implementation or delivery of the singing programme e.g., COVID-19, cultural values, different perceptions |
|  |
| **Active ingredients/programme content** – any reflections on specific aspects of programme content (e.g., the songs, room it took place in, people) that were perceived as essential to supporting symptoms of PND |
|  |

1. **Example topic guide**

Different topic guides were developed for each group of participants (e.g., referrers, management, singing leaders). The guide provided below is an example of the guide used for the focus group conducted with members of the core study management team.

1. What has been your role in the project?

Prompts: what do you feel you have contributed? What skills do you bring that you feel are important to implementing an intervention in a new cultural context?

1. What do you feel has worked well in terms of the structure of the management team?

Prompts: What’s worked and what hasn’t? Has there been any skills or expertise missing? Have there been any challenges?

**Acceptability and appropriateness of the research design in relation to future evaluation**

*This singing intervention has been part of a research project with the aim of understanding how to implement this intervention in different cultural contexts. There’s also an interest in exploring how to evaluate this programme longer-term in different countries if it can be scaled up. We are interested to know how appropriate you felt the research components were to implementing the study.*

1. How easy/difficult was it to conduct the research elements of the project?

Prompts: What management structure and support did you need? How did you find the processes of getting ethical approval? Were there any participant recruitment issues? Did anything help recruitment? What were the motivating factors for mothers to sign up?

1. What elements of the research do you feel would be appropriate or useful to evaluating future delivery of this singing intervention in other contexts? Why?

Prompts: Do you feel that anything would need to change? Why?

**Implementation strategies**

1. What has been your experience of the implementation support structures provided through the project structure?

Prompts: were the bi-weekly meetings helpful or challenging? Support from UCL? Internal support from WHO? What worked/what didn’t?

1. What internal support did you need from your own organisation in order to get this project off the ground?

Prompts: What worked/what didn’t?

*In this next section we will be talking about the delivery, structure and content of the actual singing classes themselves. The questions will be the most relevant to those who have been on-the-ground developing the singing classes in Romania and Denmark, but they are open to anyone to answer if you have reflections.*

**Acceptability, appropriateness, and feasibility of structure (how it was delivered) and content (what it was delivered)**

1. What do you think of the structure and delivery of the Music and Motherhood classes? (i.e., 1 session a week for 10 weeks)

Prompts: How *acceptable* do you think the structure and delivery is?

Do you think that the structure and delivery are satisfactory?

Are you happy with the programme being delivered in this way? Is it appropriate to your country and context?

1. What do you think of the Music and Motherhood programme content? (e.g., the songs, group activities, time for socialising)

Prompts: Do you think the content is agreeable and enjoyable for participants? Is it satisfactory?

1. How appropriate is the content and way that the intervention is structured and delivered for improving symptoms and quality of life in people with PND? Why?

Prompts: is the way the intervention is structured and delivered suitable and fit for purpose? Why?

Do you think the way the classes are structured and delivered is good? Why/why not?

**Training**

*Training on how to deliver this intervention was delivered by Breathe Arts Health Research. The next section asks questions about this training.*

1. What did you think of the structure and content of the training provided?

Prompts: Did you find it useful in your role as a manager/coordinator/researcher on the project? How was it to have a UK training provider delivering online?

1. Was there anything not covered in the training that you would have liked to have seen? If yes, what?

**Sustainability**

1. Going forward, how sustainable, in your opinion, is the delivery of these singing classes? Why?

Prompt: How easy would it be to continue running the singing classes in your country do you think?

1. If these singing classes were made more widely available such as being delivered in multiple locations and countries, what would you anticipate would be the main:

- barriers to sustained use and delivery?
- facilitators to sustained use and delivery?

1. How feasible do you think it is to get funding within your context to deliver an intervention like this in the longer term?

Prompts: what’s needed to ensure longevity of this intervention? Are there any political or cultural barriers or enablers to securing this funding?

**Costs**

1. How willing would you be to support or assist in the management or delivery of an intervention like the Music and Motherhood intervention again in the future? Why?
2. How much time and resource do you think you gave to the Music and Motherhood project to organise it?

Prompts: use of computers or printing equipment, extra staff time

1. Were there any other additional costs or resources that were drawn upon to ensure the singing intervention could be delivered smoothly that weren’t directly included in the budget to deliver the project?

**Key learnings**

1. What do you think have been the key learnings of this project?

Prompts: e.g., importance of partnerships, time needed for recruitment, importance of referral pathways, ways to document processes, particular research methods you think have been helpful

1. Overall, when thinking about the structure and delivery of the project, is there anything you felt worked particularly well?
2. And is there anything you feel didn’t go to plan? Why?
3. What do you think it is specifically about the social conditions of Romania and Denmark that meant the singing classes needed to be adapted in certain ways to meet local needs?

Prompts: e.g., structure of the healthcare system, partnerships already in place, connections to local communities, knowledge gained from previous projects

1. What would you recommend as the key structural elements required for other countries to implement this singing intervention for the first time in more new settings?

Prompts: Have you shared any particular advice with Italy and why? Any specific resources required? What partnerships are needed and why?

**Closing**

1. Do you have any other comments?
2. Do you have any questions for us?

*Thank participant(s) for contributing and for their time.*
